# Memoire on the Employment of Carbonate of Ammonia in Scarlatina

**Published:** 1843-10

**Authors:** 


Art. X.—*Mémoire sur l'Emploi du Carbonate d'Ammoniaque dans la Scarlatine.* Par le Dr. Rieken.—*Bruxelles*, 1843. 8vo, pp. 118.
*Memoir on the Employment of Carbonate of Ammonia in Scarlatina.*— By Dr. Rieken.—*Brussels*, 1843.

This memoir deserves attention, although the author appears to fall into the common error of exaggerating the virtues of the remedy which he recommends. Besides an historical notice of the use of carbonate of ammonia in scarlet fever, (for Dr. Rieken is not the original proposer of this agent,) there is an accurate account of the nature and symptoms of the disease, of the cases and periods in which carbonate of ammonia is to be had recourse to, of the ends it fulfils, and of its therapeutic modus operandi. The theories quoted on this last subject are so various, that we must refer the reader to the work itself for an account of them.

We have to add, that Dr. Rieken, though strongly impressed with a belief of the powers of carbonate of ammonia, as an anti-scarlatinal agent, by no means relies on it alone, but has also recourse to other very various and enlightened methods of opposing this formidable malady.

